# Disability independent of cerebral white matter demyelination in progressive multiple sclerosis

**DOI:** 10.1007/s00401-024-02796-w

**Published:** 2024-08-31

**Authors:** Vikas Singh, Yufan Zheng, Daniel Ontaneda, Kedar R Mahajan, Jameson Holloman, Robert J Fox, Kunio Nakamura, Bruce D Trapp

**Affiliations:** 1 Department of Neurosciences, NC30, Lerner Research Institute, Cleveland Clinic, 9500 Euclid Avenue, Cleveland, OH 44195 USA; 2 Department of Biomedical Engineering, Lerner Research Institute, Cleveland Clinic, Cleveland, OH USA; 3 Mellen Center for Treatment and Research in MS, Cleveland Clinic, Cleveland, OH USA

**Keywords:** Multiple sclerosis, Neuropathology, Neurodegeneration, MRI, Demyelination

## Abstract

The pathogenic mechanisms contributing to neurological disability in progressive multiple sclerosis (PMS) are poorly understood. Cortical neuronal loss independent of cerebral white matter (WM) demyelination in myelocortical MS (MCMS) and identification of MS patients with widespread cortical atrophy and disability progression independent of relapse activity (PIRA) support pathogenic mechanisms other than cerebral WM demyelination. The three-dimensional distribution and underlying pathology of myelinated T2 lesions were investigated in postmortem MCMS brains. Postmortem brain slices from previously characterized MCMS (10 cases) and typical MS (TMS) cases (12 cases) were co-registered with *in situ* postmortem T2 hyperintensities and T1 hypointensities. T1 intensity thresholds were used to establish a classifier that differentiates MCMS from TMS. The classifier was validated in 36 uncharacterized postmortem brains and applied to baseline MRIs from 255 living PMS participants enrolled in SPRINT-MS. Myelinated T2 hyperintensities in postmortem MCMS brains have a contiguous periventricular distribution that expands at the occipital poles of the lateral ventricles where a surface-in gradient of myelinated axonal degeneration was observed. The MRI classifier distinguished pathologically confirmed postmortem MCMS and TMS cases with an accuracy of 94%. For SPRINT-MS patients, the MRI classifier identified 78% as TMS, 10% as MCMS, and 12% with a paucity of cerebral T1 and T2 intensities. In SPRINT-MS, expanded disability status scale and brain atrophy measures were similar in MCMS and TMS cohorts. A paucity of cerebral WM demyelination in 22% of living PMS patients raises questions regarding a primary role for cerebral WM demyelination in disability progression in all MS patients and has implications for clinical management of MS patients and clinical trial outcomes in PMS. Periventricular myelinated fiber degeneration provides additional support for surface-in gradients of neurodegeneration in MS.

**Supplementary Information:**

The online version contains supplementary material available at 10.1007/s00401-024-02796-w.

## Introduction

Multiple sclerosis (MS) is an inflammatory demyelinating disease of the central nervous system that affects approximately 1 million individuals in the United States and 2.8 million individuals worldwide [36]. Most people with MS (PwMS) have a clinical course initially characterized by relapses and remissions that eventually evolves into a progressive course where relapses are rare and disability progression is continuous and irreversible [20, 33, 42, 46]. Immune-modulating therapies reduce gadolinium-enhanced cerebral white matter (WM) lesions and clinical relapses but have less robust effects on disability progression where the cause is neurodegeneration and therapeutic targets include neuroprotection, remyelination, and modulation of innate immunity [29]. Magnetic resonance imaging (MRI) of cerebral WM plays a critical role in the diagnosis and therapeutic management of individuals with relapsing-remitting MS (RRMS) and serves as an outcome measure in clinical trials designed to reduce cerebral WM demyelinating lesions [36]. MRI metrics for disease progression in progressive MS are more challenging and include abnormalities in cerebral WM and brain volume measures. While T2 lesion volume usually increases with disability progression, it accounts for less than 30% of the variance in the rate of brain atrophy, [15, 23, 38] and 45% of T2-weighted lesions in secondary progressive postmortem MS brains are myelinated [9, 16]. It remains to be determined if myelinated cerebral WM T2 hyperintensities are associated with a novel pathological process that is independent of cerebral WM demyelination.

A subset of postmortem MS brains in the Cleveland Clinic MS autopsy database (12/100) has a paucity of cerebral WM demyelination and are referred to as myelocortical MS (MCMS) based upon spinal cord and subpial cortical demyelination [43]. Despite the lack of cerebral WM demyelination, neuronal loss in 5 cortical areas was similar to that detected in postmortem MS brains with cerebral WM demyelination [43]. Global cortical atrophy in MS patients that have disability progression independent of relapse activity (PIRA) [3, 22, 34, 44, 45] and MS patients with acute clinical events with stable MRIs (ACES) [18] also raise questions regarding the role of cerebral WM demyelination as the major cause of disability in MS. While cerebral WM demyelination contributes to irreversible disability progression, it is unlikely to be the sole driving factor in progressive MS patients [4, 41]. A challenge for the MS research community is to identify causes of the permanent neurological disability that occur independent of cerebral WM demyelination. Development of MRI sequences that reliably identify myelinated cerebral WM in living MS patients would be beneficial in this regard.

The three-dimensional distribution of myelinated T2 hyperintensities in MCMS remains unknown. As T1-hypointense lesions are more sensitive for demyelination compared to T2 lesions, a multiparametric approach using T1 hypointensity thresholds may reliably distinguish myelinated from demyelinated WM lesions and possibly identify living MCMS patients [26]. There is obvious value in identifying living MCMS patients because their inclusion in a remyelinating clinical trial that focuses on cerebral T2 lesions could obscure potential benefits since T2 lesions in MCMS are not demyelinated. Identification of myelinated axonal pathology in MCMS may also identify novel treatment opportunities for progressive MS.

The present study investigates MRI and pathological changes in MCMS. We determined the three-dimensional distribution of myelinated T2 hyperintensities in MCMS and investigated the pathological correlates of myelinated T2 hyperintensities. We developed an MRI-based classifier that identified postmortem MCMS cases and applied it to baseline MRIs of 255 progressive MS patients enrolled in the Phase II, placebo-controlled, randomized, double-blind, multi-center clinical trial of ibudilast in progressive MS (SPRINT-MS, NCT01982942) [17].

## Materials and methods

### Brain tissues

Postmortem brains and spinal cords were obtained from patients with MS through the Cleveland Clinic rapid autopsy protocol [10]. Consent for *in situ* postmortem MRI and procurement of brains was obtained prior to death from living patients or family members after death. This study was approved by the Institutional Review Board of the Cleveland Clinic, Cleveland, OH, USA. Twelve previously characterized MCMS and 12 typical MS (TMS) brains were utilized for pathological studies [43]. MRI data from 2 MCMS brains are not available. Age-matched postmortem brains from 8 individuals without clinical indications or pathological evidence of neurological disease were used as controls for pathological studies. Clinical characteristics of these 30 brain donors have been described previously [43].

### Postmortem in situ brain MRI

Postmortem *in situ* MRIs were acquired prior to brain removal using standardized MRI acquisition protocols (Table 1) [16, 43]. MRI acquisition protocols for PwMS are described in Table 1. A schematic overview of the experimental workflow is provided in Fig. S1.

**Table 1:** Parameters of MRI sequences

MRI sequence	Repetition time (ms)	Inversion time (ms)	Echo time (ms)	FOV (mm)	Slice thickness (mm)	Matrix size	Scanner
Postmortem 3D FLAIR	6500	2000	403	230	5	256 × 256	Siemens 1.5T, 3T
Postmortem 3D T1w (MPRAGE)	1860	NA	2.8	240	0.94	256 × 256	
Postmortem 3D T1w (FLASH)	18	NA	5	192	1	256 × 192	
SPRINTMS 3D FLAIR	[9000, 13580]	2500	[77.0, 99.2]	[192, 256]	3	256 × 256	Siemens or GE 3T
SPRINTMS 3D T1w (FLASH)	[20, 28]	NA	6	[192, 256]	1	256 × 256	

### Postmortem MRI classifier

**Fig. 1:**
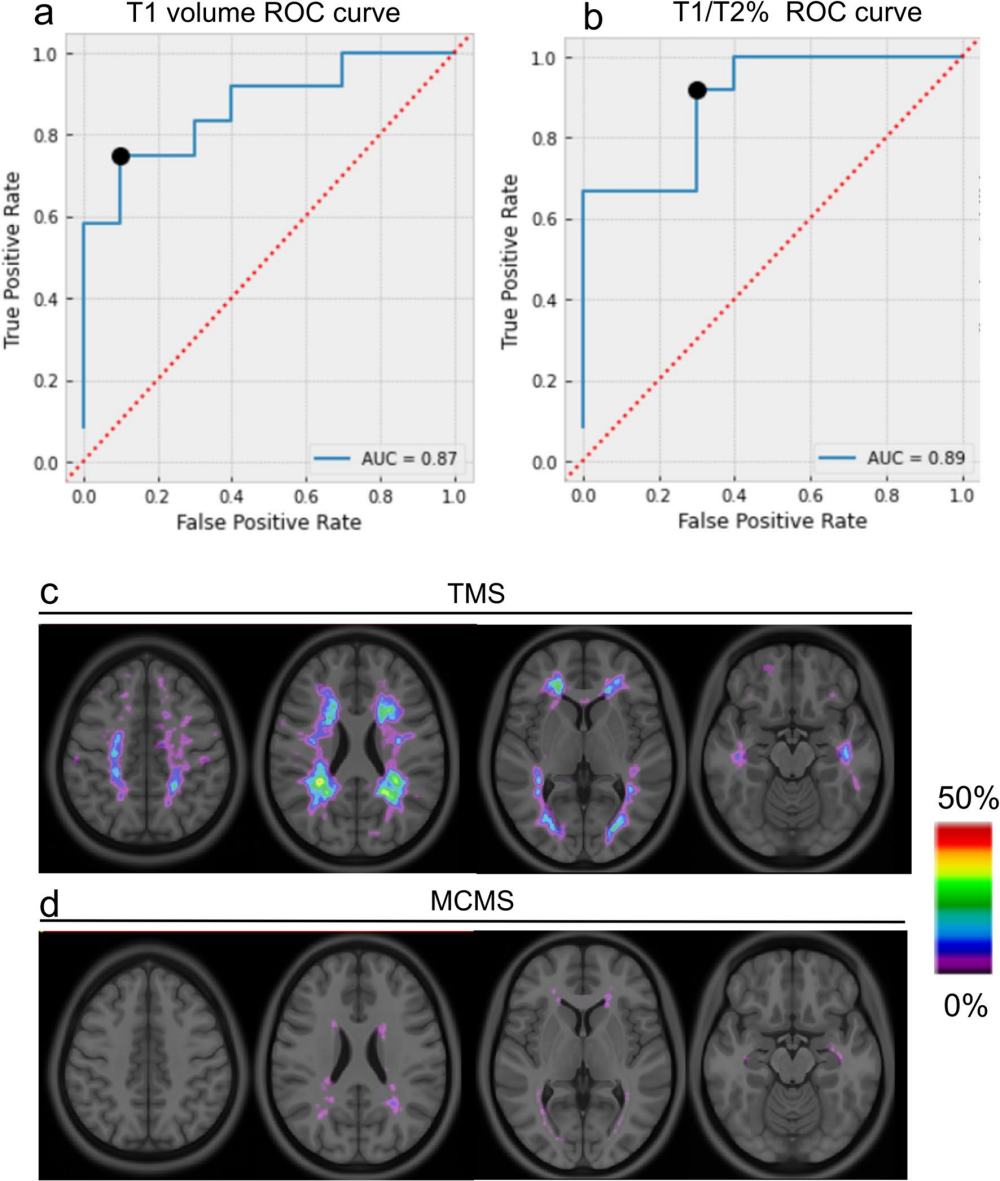
Development of an MRI-based classifier to identify the MCMS subgroup. **a-b** Receiver operating characteristic (ROC) curves for T1 volume (**a**) and T1/T2% ratio (**b**) for discriminating MCMS from TMS. **c-d** The optimal threshold for T1 hypointensities derived from ROC curves is depicted as probability maps of T1 lesions in TMS (**c**) and MCMS (**d**)

An MRI classifier was developed that differentiated MCMS and TMS based upon T2 and T1 lesion volumes using MRIs of previously-characterized postmortem cases (10 MCMS and 12 TMS brains) [43]. T2 lesions were first segmented automatically using a previously published in-house segmentation method and manually corrected when necessary [14]. For T1 lesion segmentation, mean and standard deviation (SD) intensity of cortical grey matter (GM) was calculated for each brain using the formula: Threshold value = Mean T1 intensity of GM–k (optimal scaling factor) **×** SD of GM T1 intensity. Various thresholds of these cortical GM T1 intensities were applied to the classifier (Table 2). The T1 intensity threshold that most reliably distinguished MCMS and TMS was 1.7 **×** SD below the mean cortical GM intensity. The value of k = 1.7 was identified from a receiver-operating characteristics (ROC) analysis of T1 volume and proportion of T2 lesions occupied by T1 lesions (T1/T2 %) on the same postmortem cases using an in-house deep learning method (Table 2; Fig. 1a and b). The 3D renderings of T2 hyperintensities and T1 hypointensities were created using MRIcroG [37].

**Table 2:** Testing k values to optimize T1 volumes and T1/T2% to identify MCMS

k (Scaling factor)	T1 volume				T1/T2 %			
	specificity	sensitivity	accuracy	cut-off	specificity	sensitivity	accuracy	cut-off
1	0.75	0.8	0.77	1.976	0.75	0.8	0.78	8.8
1.1	0.75	0.8	0.77	1.788	0.75	0.8	0.78	7.4
1.2	0.75	0.8	0.77	1.581	0.83	0.8	0.82	5.2
1.3	0.75	0.8	0.77	1.391	0.75	0.8	0.73	5.3
1.4	0.75	0.8	0.77	1.213	0.75	0.8	0.73	4.4
1.5	0.75	0.7	0.73	0.864	0.67	1	0.82	7.3
1.6	0.75	0.7	0.73	0.796	0.67	1	0.82	6.4
1.7	0.75	0.9	0.82	0.747	0.92	0.7	0.82	1.9
1.8	0.75	0.8	0.78	0.601	0.92	0.7	0.82	1.6
1.9	0.83	0.8	0.82	0.49	0.92	0.7	0.82	1.3
2	0.75	0.8	0.78	0.448	0.67	1	0.82	2.8

### T1 lesion probability map

Each MRI was nonlinearly registered to the International Consortium for Brain Mapping (ICBM) standard template using ANTS. The nonlinear warp was used to transform T2 lesions maps and the thresholded T1 lesion maps into the standard space where the probability maps were created. The T1 lesion probability map was blurred by Gaussian filter to account for potential spatial mismatch.

### Validation of the MRI classifier

The MRI classifier was validated on 36 uncharacterized postmortem brains with T2 lesion volumes > 2 ml (required to distinguish MCMC and TMS) and age at time of death < 65 years (upper age limit for progressive MS clinical trials) [5–7, 17]. Brain slices (24–28 cm thick) from all 36 brains were examined macroscopically for cerebral WM hyperpigmentation, measured, and expressed as cm^2^. For MCMS brains identified by the MRI classifier, MRI images were co-registered with cm-thick brain slices that contained the occipital horn of the lateral ventricle to confirm expansive T2 hyperintensities. These cm-thick brain slices were then embedded in paraffin, sectioned, and stained for myelin with proteolipid protein (PLP) antibodies. MCMS cases were identified by a paucity of macroscopic lesions in cm-thick hemispheric brain slices (total cerebral WM hyperpigmentations per hemisphere < 0.4 cm^2^)[43], the absence of demyelinated lesions in the PLP-stained hemispheric sections, the presence of demyelinated subpial cortical lesions in PLP-stained hemispheric sections, and the presence of demyelinated lesions in PLP-stained 30 µm-thick sections from spinal cord.

### Co-registration of MRI and tissue slices

Following the postmortem *in situ* MRI, the cadaver was transported to the morgue for brain and tissue collection. One cerebral hemisphere was separated and fixed in 4% paraformaldehyde for at least 9 weeks [10]. The long-fixed brain hemisphere was scanned using a high-resolution T1-weighted image in a custom-made slicing box with MRI-visible markers. After scanning, the brain tissue was sliced into 1 cm-thick coronal slices. The postmortem MPRAGE image was registered to the MPRAGE image of the fixed hemisphere using nonlinear registration tools. The postmortem MPRAGE and FLAIR image was reoriented to the location of MRI-sensitive markers. The registration between postmortem MRIs and each brain slice was visually inspected and manually corrected [16].

### MRI-pathology correlations

Postmortem *in situ* MRIs were co-registered with cm-thick coronal brain slices of 10 previously characterized MCMS cases [10]. Cm-thick brain slices containing T2 hyperintensities at the occipital horn of the lateral ventricle were cut into two 0.5 cm-thick slices. One slice was embedded in paraffin, sectioned at thickness of 10 µm, stained with PLP antibodies (clone AA3, 1:100; gift from Wendy Macklin, University of Colorado, Aurora, CO) using the avidin-biotin complex and diaminobenzidine procedure [2], and examined for demyelinated lesions in cerebral WM and cerebral cortex. Additional 10 µm-thick paraffin hemispheric sections were stained with Hematoxylin and Eosin (H&E) and analyzed for periventricular small vessel disease (SVD) using standard protocols for perivascular space area and vessel sclerotic index [30, 47]. The other 0.5 cm-thick slice was used for immunocytochemical studies comparing periventricular myelinated axonal pathology in MCMS and age-matched control brains. T2-hyperintensity-positive periventricular myelinated WM in the other 0.5 cm-thick posterior hemispheric slice was removed, sectioned at a thickness of 30 µm, double-labelled for myelin (PLP antibody, 1:100) and axons (mouse anti-phosphorylated and non-phosphorylated neurofilament antibodies, SMI31 and SMI32: 1:1000 dilutions; Bio Legend), and Alexa flour 488 anti-mouse and Alexa flour 594 anti-rat secondary antibodies (diluted 1:1000; Thermofisher Scientific), and then examined by confocal microscopy. Axon densities and axon diameters were calculated as described previously using the tile scanning feature of the Leica SP8 confocal microscope [16, 43] and measured separately at 0.75 mm intervals from the ventricular surface. Multiple nonoverlapping images at 63x magnification were stitched together to create an 27×10^4^ µm^2^ image for quantification of axonal density and axonal diameter using Image J [43]. All quantifications were performed in a blinded fashion.

### Calibration of T1 hypointensity measurements

We investigated the impact of two different 3D T1-weighted sequences on T1 hypointensity volume measurement: MPRAGE and FLASH. Thirty-five brains were scanned *in situ* with both protocols at 1mm isotropic resolution. Both images were processed independently and T1 hypointensities were segmented automatically (Fig. S2).

The T1 hypointensity volume measurements from MPRAGE and FLASH were highly correlated, and the measures were linearly calibrated using the following equation:

$${\text{T1 hypointensity volume Converted}}_{{{\text{MPRAGE}}}} = { 1}.{5327 } \times {\text{ T1 hypointensity volume}}_{{{\text{FLASH}}}}$$

### Application of the MRI classifier to PwMS

We applied the postmortem image classifier to the baseline MRIs collected in the Phase II, placebo-controlled, randomized, double-blind, multi-center clinical trial of ibudilast in progressive MS (SPRINT-MS, NCT01982942) [17]. The SPRINT-MS trial enrolled 255 people from 28 United States sites with either Primary Progressive MS or Secondary Progressive MS, age ≤65 years. Patient characteristics were mean age 58 yrs, 47% female, median disease duration 10 years, 53% with Primary Progressive MS and Expanded Disability Status Scale (EDSS) range 3.0–6.5. MRIs were obtained using either GE or Siemens 3T systems. T2 lesion volumes, T1 lesion volumes, and T1/T2% were determined according to the MRI classifier. MRI volumes of whole brain, cerebral WM, cerebral gray matter, and cortical thickness were described previously [14, 31]. MRI characteristics were correlated with clinical data obtained at study baseline [17].

### Statistical analyses

Investigators were blinded to MS classification for quantitative analyses of all datasets. Statistical analyses included unpaired *t*-tests (to compare normally distributed continuous histopathological measurements and MRI measurements between the MCMS group and the TMS or non-neurological control group), and Mann-Whitney *U* tests (to compare non-normally distributed histopathological measurements and MRI measurements between the MCMS group and the TMS or non-neurological control group). For *in vivo* MRI analyses, *t*-tests on baseline variables and calculations of linear correlation coefficients (to evaluate the associations between T1 lesion volume and EDSS) were performed. Welch corrections were used if a dataset failed to show equal variance in an *F*-test. A Chi square test was used for categorical datasets. For all statistical analyses, two-sided P values of 0.05 or less were considered significant.

## Results

### MRI classifier

Using MRI sequences and brain slices obtained from previously characterized postmortem MCMS (10 cases) and TMS (12 cases) brains, a classifier that uses T1 hypointensity volume and the percentage of T2 hyperintensity volume occupied by T1 hypointensity volume (T1/T2% ratio) was developed. Since T1 hypointensities vary within and between brains, we systematically obtained T1 thresholds that differentiated MCMS from TMS brains (Fig. 1). The ROC curves were obtained from different T1 hypointensity thresholds and T1/T2% ratios. The optimal (Youden’s) cut point with the greatest sensitivity and specificity to discriminate MCMS from TMS cases was a threshold T1 lesion volume < 0.75 ml (specificity 75%, sensitivity 90%, and accuracy 82%) and T1/T2 % ratio < 1.9% (specificity 92%, sensitivity 70%, and accuracy 82%) (Fig. 1a and b, Table 2). The area under the curve (AUC) that discriminated MCMS from TMS was 0.87, (95% CI 0.64–0.96) for T1 hypointensity volume and 0.89 (95% CI 0.58–0.94) for T1/T2% ratio (Fig. 1a and b). All 22 brains (10 MCMS and 12 TMS) had T2 volumes > 2.0 ml. The spatial distributions of T1-weighted lesions were different in probability maps of TMS and MCMS brains (Fig. 1c and d). The T1 lesion probability in lesion voxels was higher in TMS (0-46%) compared to MCMS (0-16%) (Fig. 1c and d). The MRI classifier thus reliably distinguished previously-characterized MCMS and TMS cases.

### Three-dimensional renderings of T2 and T1 lesions in TMS and MCMS

**Fig. 2:**
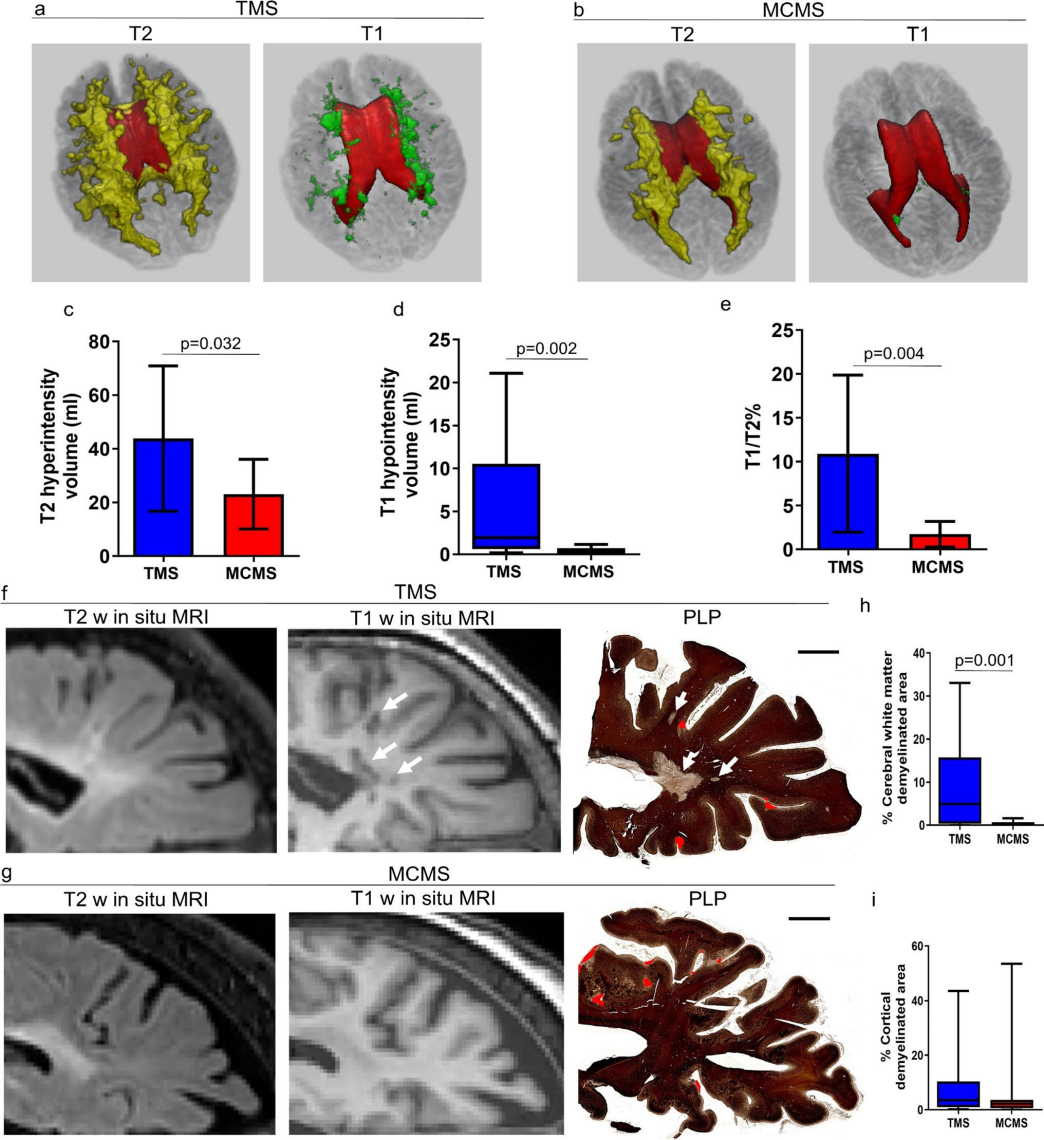
Three-dimensional distribution of T2 hyperintensities and T1 hypointensities in typical and myelocortical postmortem MS brains. **a-b** Three-dimensional maps of T2 hyperintensities (yellow) and T1 hypointensities (green) in postmortem TMS (**a**) and MCMS brains (**b**). T2 hyperintensities in both TMS and MCMS brains had a contiguous periventricular distribution that expanded at the occipital horn. T1 hypointensities were abundant in TMS (**a**, green) and were rare in MCMS (**b**, green). **c-e** Quantification of T2 volume (**c**), T1 volume (**d**), and T1/T2% ratio (**e**) in TMS and MCMS brains. **f-g** Postmortem T2-weighted and T1-weighted images co-registered with PLP-stained hemispheric brain sections from TMS (**f**) and MCMS (**g**) confirmed demyelination of T1 hypointensities in TMS (white arrows) and the paucity of WM demyelination in MCMS. Cortical demyelination (red) was present in PLP-stained sections from TMS and MCMS brains (*scale bar = 2 mm*). **h-i** Quantification of the percentages of cerebral WM demyelinated area (**h**) and cortical demyelinated area (**i**) in PLP-stained sections. N = 12 TMS and 12 MCMS postmortem brains

Using the classifier described above, three-dimensional renderings of T2 and T1 lesions were visualized in previously characterized postmortem brains from the 10 MCMS and 12 TMS cases [43]. The majority of T2 hyperintensities in both MCMS and TMS cases had a contiguous periventricular distribution that expanded at the occipital horn of the lateral ventricles (Fig. 2a and b). TMS brains also had focal WM T2 hyperintensities not contiguous with periventricular T2 hyperintensities (Fig. 2a). Total T2-hyperintensity volume was significantly greater in TMS (43.81 ml [SD 27.06]) compared to MCMS (23.08 ml [SD 12.98]; P = 0.032; Welch corrected unpaired *t*-tests; Fig. 2c). In MCMS, T1 hypointensities were rare, relatively small, and located near the surface of the lateral ventricles (Fig. 2b). Mean total T1 hypointensity volumes were significantly lower in MCMS (0.265 ml [IQR 0.067–0.722]) compared to TMS (1.95 ml [IQR 0.63–10.52]; P = 0.0026; Mann-Whitney *U* test; Fig. 2d). Similarly, the percentages of T1 hypointensity volume within T2 lesions were significantly lower in MCMS (1.736% [SD 1.462]) compared to TMS (10.92% [SD 8.945]; P = 0.0045; Welch corrected unpaired *t*-tests; Fig. 2e). Myelinated T2 hyperintensities in postmortem MCMS brains have a contiguous periventricular distribution that expands at the occipital horn of the lateral ventricle.

Since 3D rendering consistently show expansion of T2 hyperintensities at the occipital horns of the lateral ventricles (Fig. 2a and b), we co-registered posterior cm-thick hemispheric brain slices (from the 12 TMS cases and 10 MCMS cases) with T2 hyperintensities and T1 hypointensities (Fig. 2f and g). The hemispheric slices were processed for immunocytochemical detection of myelin (Fig. 2f and g). In TMS cases, T1 hypointensities co-localized within T2 hyperintensities that were demyelinated in the stained hemispheric section (Fig. 2f). In contrast, T1 hypointensities and demyelination were not prominent features of periventricular T2 lesions in MCMS cases (Fig. 2g). The percent area of cerebral WM demyelination was significantly lower in MCMS (0.076% [IQR 0.001–0.710]) than in TMS cases (4.906% [IQR 0.351–15.73]; P = 0.002; Mann-Whitney *U* test; Fig. 2h), while the percent areas of subpial cortical demyelination were similar (Fig. 2i). These data establish a paucity of cerebral WM demyelination associated with the occipital horn of the lateral ventricle in MCMS cases.

### Neuropathological changes in the periventricular region

**Fig. 3:**
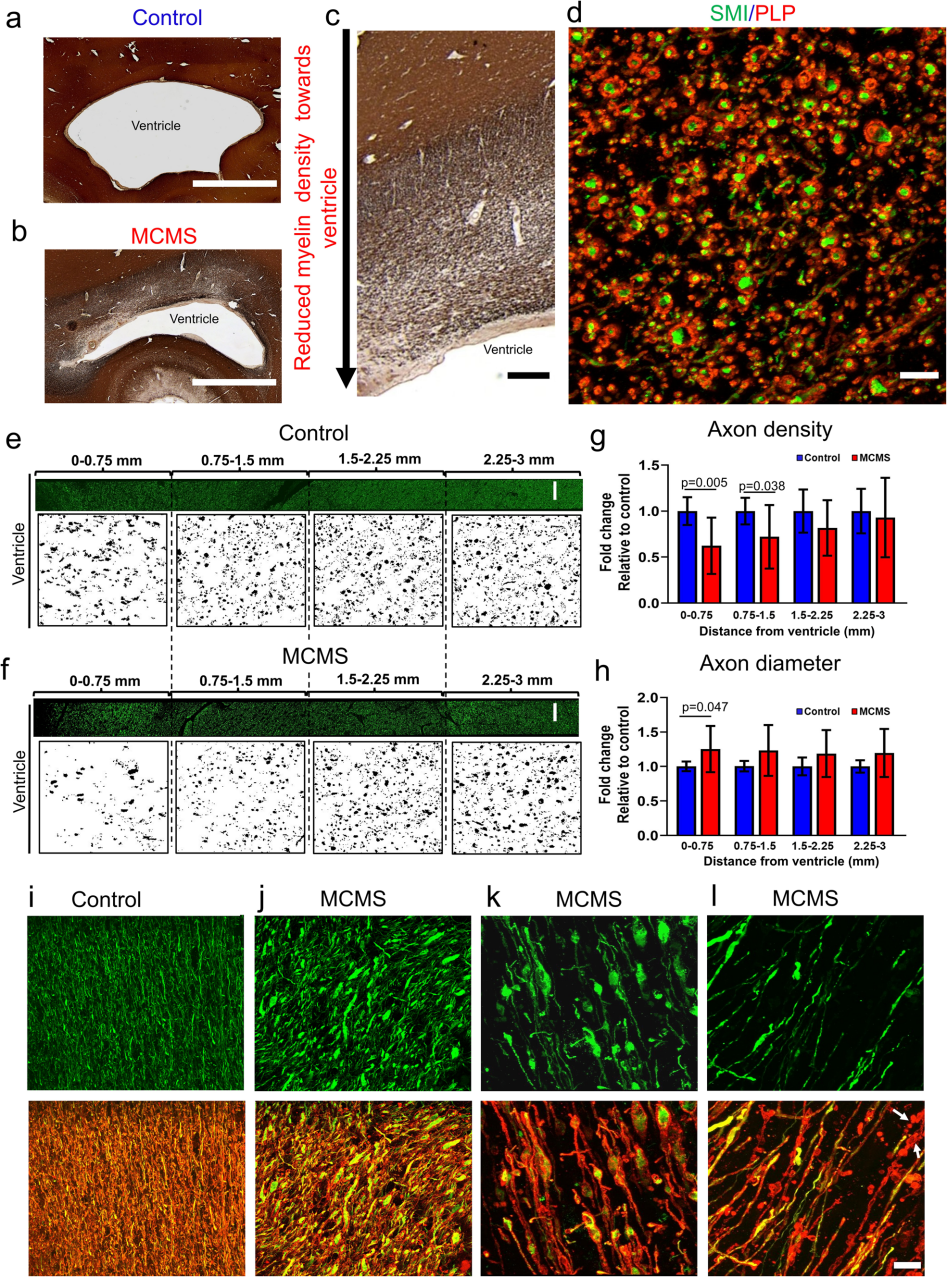
Degeneration of myelinated axons in MCMS periventricular WM.** a-c** Comparison of myelin staining (PLP antibody) in periventricular regions of control (**a**) and MCMS (**b**) brain sections (*scale bar = 2 mm*) obtained from the occipital horn of the lateral ventricle identified reduced myelin fiber density in MCMS sections (**b, c**) (c, *scale bar = 1 mm*). **d** Confocal analyses of PLP (red) and neurofilament (green) staining indicated that axons were surrounded by PLP-positive myelin in both control and MCMS sections (*scale bar = 20 µm*). **e-f** Axons in cerebral WM of non-neurological control (**e**) and MCMS (**f**) sections at 0.75 mm intervals from the ventricular surface (*scale bar = 100 µm*). **g-h** Axonal densities were decreased (**g**) and axonal diameters were increased (**h**) in MCMS segments compared to control segments. **i-l** Upper panels compare neurofilament-stained axons in control (**i**) and MCMS (**j-l**) periventricular WM. Lower panels indicate that all neurofilament-positive axons (green) are surrounded by PLP-positive myelin (red). Myelinated fibers in control sections have small diameters (**i**). MCMS sections contained axonal swellings (**j**), axonal ovoids (**k**), and myelinated axon degeneration (**l**, myelin debris without axons, arrows) (*i-l, scale bar = 30  µm*). Lower panels are double-labelled with PLP and neurofilament. **g-h** N = 8 control cases and 10 MCMS cases

While periventricular demyelination was not a prominent feature in 10 µm-thick hemispheric sections from MCMS cases (Fig. 2b), reduced myelin density was apparent in periventricular regions of the occipital horn (Fig. 3a-c). Therefore, we compared myelinated axon densities and myelinated axon diameters in periventricular regions of MCMS and aged-matched control brains double-labelled for myelin and axons. All axons in control and myelocortical periventricular WM were surrounded by PLP-positive myelin (Fig. 3d). Compared to age-matched control sections, MCMS sections contained a surface-in gradient of reduced myelinated axon densities (Fig. 3e-g) and increased myelinated axonal diameters (Fig. 3h). When examined at higher magnification (Fig. 3i-l), several patterns of myelinated axonal pathology were detected in periventricular regions of MCMS cases. Compared to control cases where periventricular myelinated axons had small diameters (Fig. 3i), myelocortical myelinated fibers had increased axonal diameters (Fig. 3j), focal axonal swellings (Fig. 3k), and discontinuous myelin ovoids without axons (Fig. 3l). Ongoing myelinated axonal degeneration was associated with decreased myelinated fiber density in periventricular WM in MCMS. These data establish a surface-in gradient of myelinated axon degeneration in the occipital horn from MCMS cases.

### Validation of the MRI classifier in postmortem cases

**Fig. 4:**
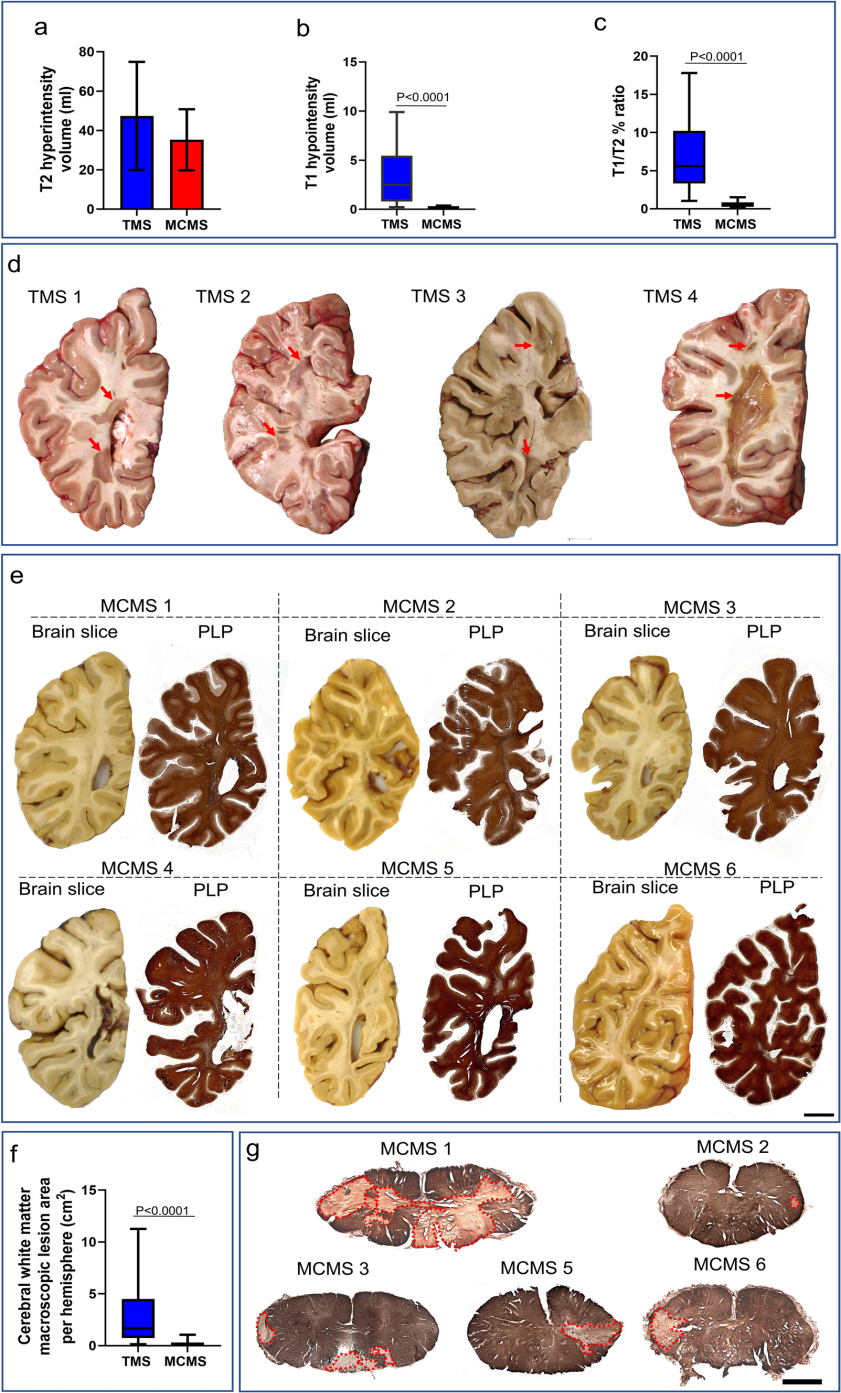
Validation of the classifier in 36 uncharacterized postmortem MS brains. **a-c** The classifier identified 29 TMS and 7 MCMS cases. MCMS brains had T2LV > 2 ml (**a**), T1 lesion volume < 0.75 ml (**b**) and T1/T2% ratio < 1.9 (**c**). **d** Representative images of cm-thick brain slices from 4 TMS brains. Macroscopic WM discolorations (arrows) indicate demyelination. **e** Co-registration of cm-thick hemispheric brain slices and myelin-stained sections from 7 MCMS cases identified by the classifier failed to detect WM hyperpigmentation in cm-thick slices nor demyelination in PLP-stained sections in 6 cases *(scale bar = 10 mm)*. One MCMS case had WM hyperpigmentation volume > 0.04 cm^2^ and was identified as an MCMS false-positive. **f** Comparison of cerebral WM hyperpigmentation between TMS and MCMS brains. **g** Spinal cord demyelination was present in 5 MCMS cases (spinal cord was not available from one case; *scale bar = 200 µm)*

We then applied the classifier to 36 uncharacterized postmortem brains (Fig. 4). Seven cases with T1 lesion volume < 0.75 ml and T1/T2% ratio < 1.9% were identified as possible MCMS cases compared to twenty-nine as possible TMS (Table 3 and Table S1). Total T2 lesion volume was similar between TMS and MCMS (Fig. 4a), whereas T1 lesion volume (Fig. 4b: TMS 2.49 ml [IQR 0.80–5.45] vs MCMS 0.12 ml [0.07–0.32]; P < 0.0001; Mann-Whitney *U* test) and T1/T2% ratio (Fig. 4c: TMS 5.57 ml [IQR 3.32–10.22] vs MCMS 0.42 ml [0.26–0.83]; P < 0.0001; Mann-Whitney *U* test) was significantly greater in TMS postmortem brains. Brain atrophy measures were similar across TMS and MCMS (Table 3). The paucity of cerebral WM hyperpigmentation (< 0.4 cm^2^) [43] in cm-thick hemispheric brain slices and the lack of cerebral WM lesions in PLP-stained paraffin sections from hemispheric brain slices (containing the occipital horn of the lateral ventricle) indicated that six of these seven cases were MCMS (Fig. 4d and e). Pathological examination identified one MCMS as TMS (Fig. S3). The classifier differentiated between MCMS and TMS with a sensitivity of 85.7%, specificity of 96.5%, and accuracy of 94.4%. The area of cerebral WM hyperpigmentation in cm-thick brain slices (28-32 per brain) was significantly lower in MCMS (0.15 cm^2^ [IQR 0.00–0.30]) compared to TMS cases (1.67 cm^2^ [IQR 0.74–4.49]; P < 0.0001; Mann-Whitney *U* test; Fig. 4f). To meet the pathological criteria for MCMS requires spinal cord demyelination. Therefore, we stained MCMS spinal cord segments for myelin with PLP antibodies. Spinal cords from 5 of the MCMS cases contained WM demyelinated lesions (Fig. 4g; spinal cord from one MCMS was not available). When applied to 36 uncharacterized postmortem brains, the classifier identified 6 MCMS cases.

**Table 3:** The clinical, imaging, and pathological characteristics of the postmortem validation cohort after stratification

	TMS	MCMS	P value
Count	29	7	NA
Age (years), median (IQR)	55 (50-60)	56 (52-61)	0.52
Female %	62%	85.7%	0.23
Primary progressive/secondary progressive/relapsing remitting	8/17/4	2/5/0	NA
T2 lesion volume (ml), median (IQR)	47.40 (27.45)	35.24 (15.51)	0.27
T1 lesion volume (ml), median (IQR)	2.49 (0.80-5.45)	0.12 (0.07-0.32)	< 0.0001
T1/T2 %, median (IQR)	5.57 (3.32-10.22)	0.42 (0.26-0.83)	< 0.0001
Brain parenchymal fraction, mean (SD)^a^	0.72 (0.08)	0.72 (0.08)	0.96
White matter fraction, median, (IQR)^b^	0.44 (0.41-0.47)	0.42 (0.3-0.48)	0.49
Grey matter fraction, mean (SD)^c^	0.28 (0.05)	0.31 (0.04)	0.21
Expanded disability status scale, median (IQR)	8 (7.2-9.0)	7.5 (7-8)	0.19
Disease Duration (years), mean (SD)	22 (9)	24 (5)	0.77
Cerebral white matter macroscopic lesion area per hemisphere (cm^2^), median (IQR)	1.67 (0.74–4.49)	0.15 (0.00-0.3)	< 0.0001

Data missing for ^a^BPF (TMS n = 1)

^b^WMF (TMS n = 1 and MCMS n = 1)

^c^GMF (TMS n = 1 and MCMS n = 1)

### MRI classifier applied to PwMS

**Fig. 5:**
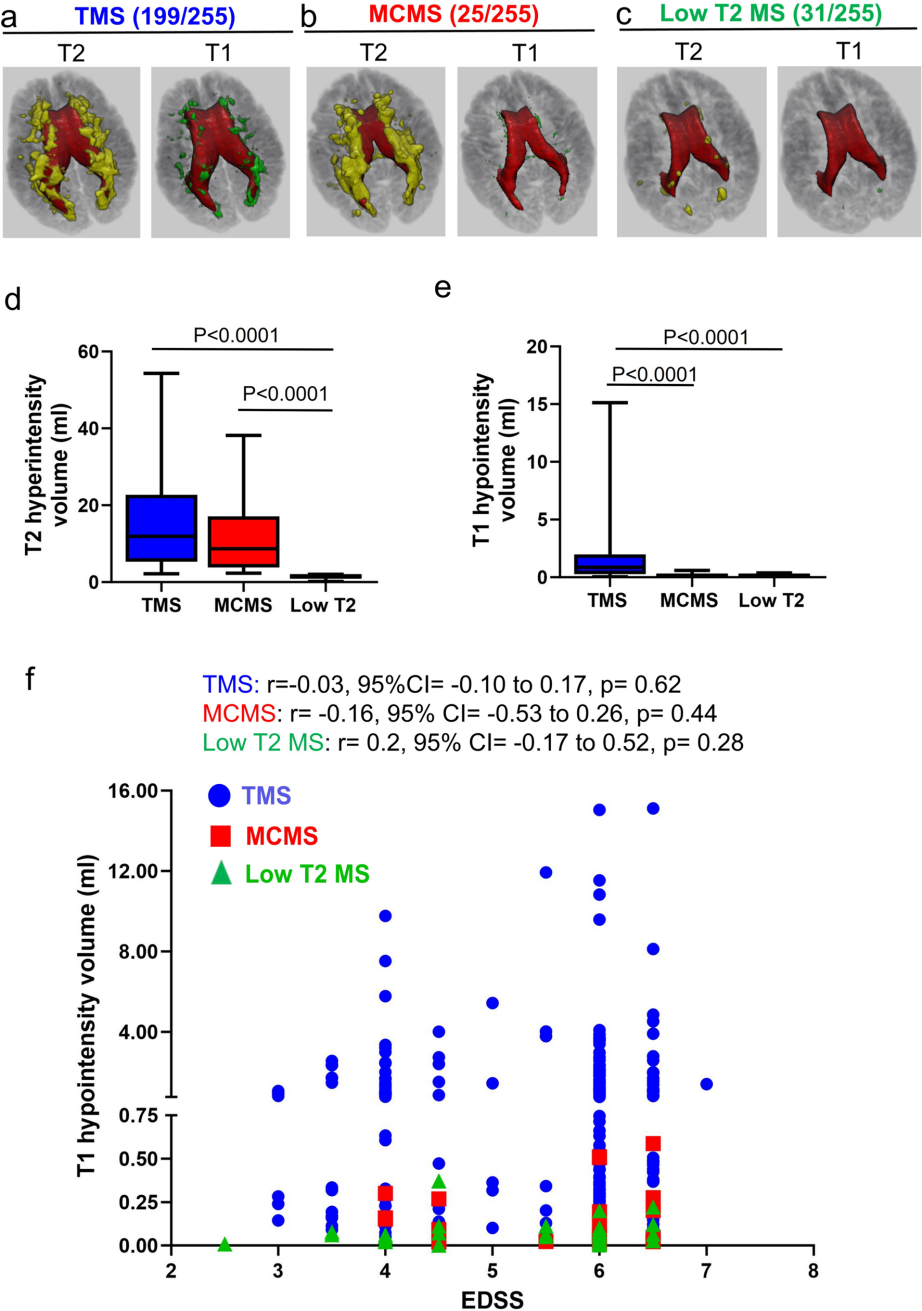
MRI classifier applied to 255 MS participants enrolled in the SPRINT-MS trial. **a-c** The MRI classifier identified 199 TMS cases (**a**), 25 MCMS cases (**b**), and 31 cases with T2 volume < 2 ml and a paucity of T1 hypointensities (**c**, Low T2). **d-e** Comparison of T2-hyperintensity volume (**d**) and T1-hypointensity volume (**e**) in TMS, MCMS, and Low T2 brains. **f** Spearman’s correlation between T1-hypointensity volume and EDSS across 199 TMS, 25 MCMS, and 31 Low T2 MS

The imaging classifier was then applied to the baseline MRIs of 255 progressive MS patients enrolled in the SPRINT-MS trial. Patient characteristics include mean age 58 yrs, 47% female, median EDSS 6.0, median disease duration 10 years, and 53% with Primary Progressive MS. Based upon quantitative measures of T2 hyperintensities and T1 hypointensities, the classifier identified 199 TMS patients (Fig. 5a) and 25 MCMS patients (Fig. 5b). Thirty-one SPRINT-MS patients did not meet inclusion criteria for distinguishing MCMS and TMS because they had < 2 ml of total T2 lesion volume (Low T2 MS group; Fig. 5c). Total T2-hyperintensity volumes were similar between TMS and MCMS (Table 4 and Fig. 5d) whereas T1 hypointensity volumes were significantly greater in TMS patients (TMS 0.86 ml [IQR 0.28–1.97] vs. MCMS 0.066 ml [0.027–0.23]; P < 0.0001; Mann-Whitney *U* test) (Table 4 and Fig. 5e). Compared to TMS, the low T2 MS group had significantly lower T1 hypointensity volumes (Table 4 and Fig. 5e, TMS 0.86 ml [IQR 0.28–1.97] vs. low T2 0.061 ml [0.025–0.086]; P < 0.0001; Mann-Whitney *U* test). In summary, 78% of SPRINT-MS patients were classified as TMS (T2 hyperintensity volume > 2 ml, T1 hypointensity volume > 0.75 ml and T1/T2% > 1.9% or T1 hypointensity volume > 0.75 ml and T1/T2% < 1.9% or T1 hypointensity volume < 0.75 ml and T1/T2% > 1.9%), 10% were classified as MCMS (T2 hyperintensity volume > 2 ml, T1 hypointensity volume < 0.75 ml; T1/T2% < 1.9%) and 12% were classified as Low T2 (T2 hyperintensity volume < 2 ml and a paucity of T1 hypointensities averaging 0.061 ml).

**Table 4:** The baseline clinical and imaging characteristics of SPRINT-MS trial participants after stratification

	TMS	MCMS	Low T2 MS	TMS vs MCMS, P value	TMS vs low T2 MS, P value	MCMS vs low T2 MS, P value
Count	199	25	31	NA	NA	NA
Age (years), mean (SD)	55.7(7.3)	56.7(6.5)	53.8(7.7)	0.63	0.19	0.2
Female %	54.3%	44.0%	54.8%	0.49	1.0	0.59
Primary progressive/secondary progressive	98/101	14/11	22/9	0.67	0.04	0.38
Brain parenchymal fraction, Median (IQR)	0.805 (0.788–0.821)	0.804 (0.783–0.819)	0.829 (0.811–0.845)	0.96	< 0.0001	0.001
T2 lesion volume (ml), median (IQR)	11.95 (5.32–22.71)	8.70 (3.82–17.13)	1.55 (1.17–1.78)	0.26	< 0.0001	< 0.0001
T1 lesion volume (ml) median (IQR)	0.86 (0.28–1.97)	0.066 (0.027–0.23)	0.061 (0.025–0.086)	< 0.0001	< 0.0001	0.18
T1/T2 % median (IQR)	6.15 (3.94–11.31)	1.00 (0.54–1.69)	3.87 (1.75–7.01)	< 0.0001	0.0006	< 0.0001
White matter fraction, median (IQR)	0.345 (0.331–0.357)	0.351 (0.334–0.367)	0.356 (0.347–0.364)	0.26	0.0024	0.24
Grey matter fraction, mean (SD)	0.458(0.020)	0.453(0.022)	0.472(0.016)	0.25	0.0004	0.0006
Cortical thickness (mm), median (IQR)	3.402 (3.250–3.539)	3.395 (3.264–3.559)	3.569 (3.470–3.701)	0.91	< 0.0001	< 0.0001
Lesional MTR, median (IQR)^a^	38.89 (36.94–41.20)	42.65 (39.81–44.55)	41.35 (39.13–42.88)	< 0.0001	0.0003	0.21
EDSS, median (IQR)	6(3~7)	6(4~6.5)	6(2.5~6.5)	0.08	0.359	0.04
Disease duration (Years), median (IQR)	11 (0~41)	9 (0~34)	7 (0~29)	0.66	0.059	0.34
Timed 25-ft walk (Sec), median (IQR)	9.55 (6.85–16.63)	11.85 (7.65–28.45)	8.5 (6.13–11.7)	0.59	0.60	0.16
9-hole peg test (Sec), median (IQR)	29.83 (25.09–40.27)	35.43 (29.9–45.33)	24.53 (21.03–27.18)	0.12	0.0008	< 0.0001
Symbol digit modalities test- no of correct answers, Mean (SD)	42.08 (14.61)	39.64 (14.23)	48.5 (11.25)	0.69	0.057	0.057

^a^MTR magnetization transfer ratio

^b^EDSS expanded disability status scale

TMS and MCMS participants had similar disability scores (EDSS), disease duration, reduced whole-brain, WM, and GM volumes, and similar degrees of cortical thinning (Table 4). The low T2 group had lower disability scores and less brain atrophy compared to TMS and MCMS (Table 4). There was no correlation between EDSS and T1 hypointensity volume (Fig. 4f) in TMS (r = 0.03), MCMS (r = 0.16), or low T2 (r = 0.20) patients (Table 4 and Fig. 5f). The SPRINT-MS trial enrolled 134 PPMS and 121 SPMS participants: 50% percent of TMS, 56% of MCMS, and 71% of low T2 participants were identified as PPMS (Table 4).

## Discussion

We developed an MRI classifier that reliably identified previously-characterized postmortem MCMS brains with a paucity of cerebral WM demyelination by co-registrations of the MRIs and myelin pathology in tissue sections. The classifier was validated in 36 uncharacterized postmortem MS brains and identified 6 MCMS cases with 94% accuracy by macroscopic assessment of brain slices and myelin staining of tissue sections. An advantage of the classifier described here is that it utilizes imaging sequences that are routinely performed in the clinic and in MS clinical trials. The imaging classifier identified a contiguous periventricular distribution of myelinated T2 hyperintensities in postmortem MCMS brains that expanded at the occipital horn of the lateral ventricles where a surface-in gradient of myelinated fiber degeneration was detected. This adds to the growing evidence that a surface-in mechanism of neurodegeneration is operating in MS [27, 28]. When applied to 255 PMS participants enrolled in SPRINT-MS, the classifier identified 25 MCMS cases. These MCMS patients had similar disability scores (EDSS) and cortical thinning as TMS patients, supporting the concept that neurological disability and cortical atrophy can occur independent of cerebral WM demyelination. By leveraging postmortem and clinical MRI signatures, our studies support the utilization of an MRI classifier for establishing inclusion criteria and outcome measures for future PMS clinical trials. The clinical implications of this work are important, as the absence of cerebral WM demyelination was a relatively common finding, and inclusion of these patients in potential remyelination trials may dilute power. The use of the classifier to enrich study populations with demyelination would be a major added value for recruitment in remyelination trials or in post hoc analysis of already-completed trials.

This study supports the concept that MS is characterized by an insidious neurodegenerative process evident by widespread cortical atrophy, which can occur independent of superimposed and variable cerebral WM demyelination [4, 24]. Longitudinal studies of two RRMS populations concluded that 80-90% of overall disability progression occurred independently of relapse activity (PIRA) [3, 22, 34, 44, 45]. New focal T2 or Gadolinium-enhancing lesions need not accompany all clinical relapses (ACES) [18]. Our identification of postmortem brains from severely disabled MS cases with a paucity of cerebral WM demyelination provides pathological support of disability independent of significant cerebral WM demyelination. Demyelination of spinal cord white matter, however, is likely to contribute to disability progression in MCMS.

Myelinated T2 hyperintensities have contiguous periventricular distribution in MCMS brains that expands at the occipital pole of the lateral ventricle where a gradient of myelinated axonal loss and degenerating myelinated internodes peaks at the ventricular surface and decreases with distance from this surface. Such a surface-in mechanism of MS pathogenesis is not novel and has been described for the pial surface of the cerebral cortex [27] and third ventricle of the thalamus [28]. Meningeal inflammation in cortical sulci has been proposed as a source of CSF immune-related soluble factors that diffuse into the cortex and cause subpial demyelination of cortical layers I–III [1, 21, 27]. The surface-in gradient at the occipital horn of the lateral ventricle described here, however, differs from that in the cortex in that it does not induce periventricular WM demyelination, targets the integrity of the myelin-axon unit, and its surface is comprised of ependymal cells. Pathological and molecular characterization of ependymal cells in postmortem MCMS brains may help to identify mechanisms of periventricular myelinated axon loss.

Periventricular myelinated T2 hyperintensities are also present in TMS brains. It remains to be determined whether a surface-in mechanism of myelinated axonal degeneration could be segregated from periventricular myelinated axon loss caused by periventricular or cerebral WM demyelination. In either event, the characterization of periventricular axonal degeneration in MCMS identifies a novel disease mechanism that has the potential to contribute to neurological decline in MS and possibly provide a therapeutic target for mitigating neurodegeneration.

Cerebral WM T2 hyperintensities have been associated with SVD. The pathological hallmarks of SVD, lacunar infarcts and microbleeds [43], and small vessel pathology (Fig. S4) are not features of MCMS cerebral WM. However, the possibility that age-related changes other than SVD contribute to periventricular WM changes cannot be excluded. While WM demyelination and SVD are not responsible for periventricular myelinated axonal degeneration in MCMS, subpial cortical and spinal cord demyelination could play a role. Spinal cord pathology is an unlikely source of periventricular myelinated axon pathology as neither motor nor sensory spinal cord axons transverse periventricular WM. The extent of cortical demyelination was similar in posterior regions of MCMS and TMS (Fig. 2i), while a previous study of parietal sections reported greater cortical demyelination in TMS [43]. In addition to a surface-in gradient, Wallerian degeneration due to cortical neuronal loss could contribute to periventricular myelinated fiber loss.

Our data supports disability with minimal cerebral WM demyelination in 22% of progressive MS patients enrolled in SPRINT–MS. The best correlates of disability at all stages of MS (clinically isolated syndrome through SPMS) are GM atrophy and cortical thinning [11, 12, 19]. Neurological disability (Table 3) and cortical neuronal loss were similar in postmortem MCMS and TMS brains [43] and neurological disability and cortical thinning were similar in TMS and MCMS participants enrolled in SPRINT-MS (Table 4). These data support the concept that cortical neuronal loss and cortical thinning can occur independent of cerebral WM demyelination.

The MS classifier distinguished pathologically confirmed postmortem MCMS and TMS cases with an accuracy of ~94%. The percentage of MCMS cases identified by the MRI classifier was similar in postmortem and SPRINT-MS cohorts. SPRINT-MS trial participants, however, had less T1 and T2 lesion volumes, reflecting their lower age, shorter disease duration, and perhaps greater access to disease-modifying therapies. Twelve percent of SPRINT-MS patients had a paucity of T2 and T1 abnormalities. The finding of a group of patients with low T2 and T1 abnormalities is not surprising, as there have long been descriptions of patients with lower brain lesion loads, particularly in those with PP forms of MS [8] or spinal cord-predominant forms of the disease [32]. SPRINT-MS patients with low T2 lesions were predominantly PPMS (22/31) and compared to TMS had significantly less MRI measures of atrophy and performed better on neural performance testing (Table 4). In summary, patients with low T2 lesion volumes appeared to have a milder form of MS. It remains to be determined if they form a distinct entity or simply reflect the lower end of the biological, clinical, and/or radiological variability that occurs in MS.

There are limitations to this study. First, it is a cross-sectional study of postmortem brains from the Cleveland Clinic and living progressive MS patients enrolled in the SPRINT-MS clinical trial. Pathological findings need to be confirmed in other autopsy cohorts and future longitudinal studies of PwMS are needed to verify MRI-based subclasses at earlier stages of MS and investigate how MRI subclasses respond to different therapeutics in clinical trials. T1 hypointensities are less specific to myelin than advanced MRI sequences such as myelin water imaging [25], magnetization transfer imaging [39], and ultrashort echo imaging [40]. While our inclusion of T1 intensity thresholds identified MCMS and TMS with an accuracy of 94%, application of these advanced MRI sequences may further optimize classifier performance. Features other than demyelination could influence the classifier. In addition to demyelination, acute lesions have increased cellular [35] and water content [13], while chronic lesions have variable degrees of tissue damage [16]. Intracortical and subpial cortical lesions may influence values of cortical T1 hypointensity thresholds, although the effect is likely small. The inclusion of MRI data acquired with different protocols and pulse sequences may influence classifier performance. Spinal cord MRI data was not included in the SPRINT-MS trial. The addition of spinal cord MRIs in future MS clinical trials would help to address the role of spinal cord disease in MS subgroups. Using pathological specimens to inform changes in people living with MS has inherent challenges; we have tried to mitigate these by 1) using MRI performed very close to the time of death, and 2) recapitulating pathology data in a clinical trial dataset. Our findings, however, may not apply uniformly to all stages of MS, particularly at early stages where the factors governing the disease process may differ.

Overall, accumulating evidence raises questions as to cerebral WM demyelination being the major cause of permanent neurological disability in all individuals with MS and supports the concept that global cortical atrophy also drives disability progression in MS. The implications of transforming a fundamental aspect of MS pathogenesis that has dominated MS research for decades are daunting and will require a better understanding of disease mechanisms that drive cortical atrophy. In this regard, pathological studies support a surface-in gradient of neurodegeneration at the pial surface of the cerebral cortex, 3^rd^ ventricle of the thalamus, and the posterior horn of the lateral ventricle, implicating a cerebrospinal fluid (CSF) -associated mechanism of neurodegeneration. Implications of the SPRINT-MS data raise the possibility that an imaging classifier can be used to stratify MS patients in clinical trials and eventually to facilitate personalized application of MS therapies.

### Supplementary Information

Below is the link to the electronic supplementary material.

Supplementary file1 (PDF 768 KB)

## Data Availability

The data that support the findings described in this study are available from the corresponding author, BDT, upon reasonable request and execution of an institutional data transfer agreement.
