# Supplementary material for: Disability independent of cerebral white matter demyelination in progressive multiple sclerosis
Source: Acta Neuropathol. 2024 Aug 31;148(1):34. doi: 10.1007/s00401-024-02796-w (PMC11365858; doi:10.1007/s00401-024-02796-w)
Supplement: Supplementary file 1 — Supplementary file1 (PDF 768 KB) [file 401_2024_2796_MOESM1_ESM.pdf]

Supplemental data

Disability independent of cerebral white matter demyelination in progressive multiple sclerosis

Vikas Singh[1], Yufan Zheng[2], Daniel Ontaneda[3], Kedar R Mahajan[1,3], Jameson Holloman[1,3], Robert J Fox[3], Kunio Nakamura[2], and Bruce D Trapp[1]

Affiliations

[1]Department of Neurosciences, Lerner Research Institute, Cleveland Clinic, Cleveland OH

[2]Department of Biomedical Engineering, Lerner Research Institute, Cleveland Clinic, Cleveland OH

[3]Mellen Center for Treatment and Research in MS, Cleveland Clinic, Cleveland, OH

Correspondence to: Dr. Bruce D Trapp, PhD, NC30, Department of Neurosciences, Lerner Research Institute, Cleveland Clinic, 9500 Euclid Avenue, Cleveland, OH, 44195; trappb@ccf.org

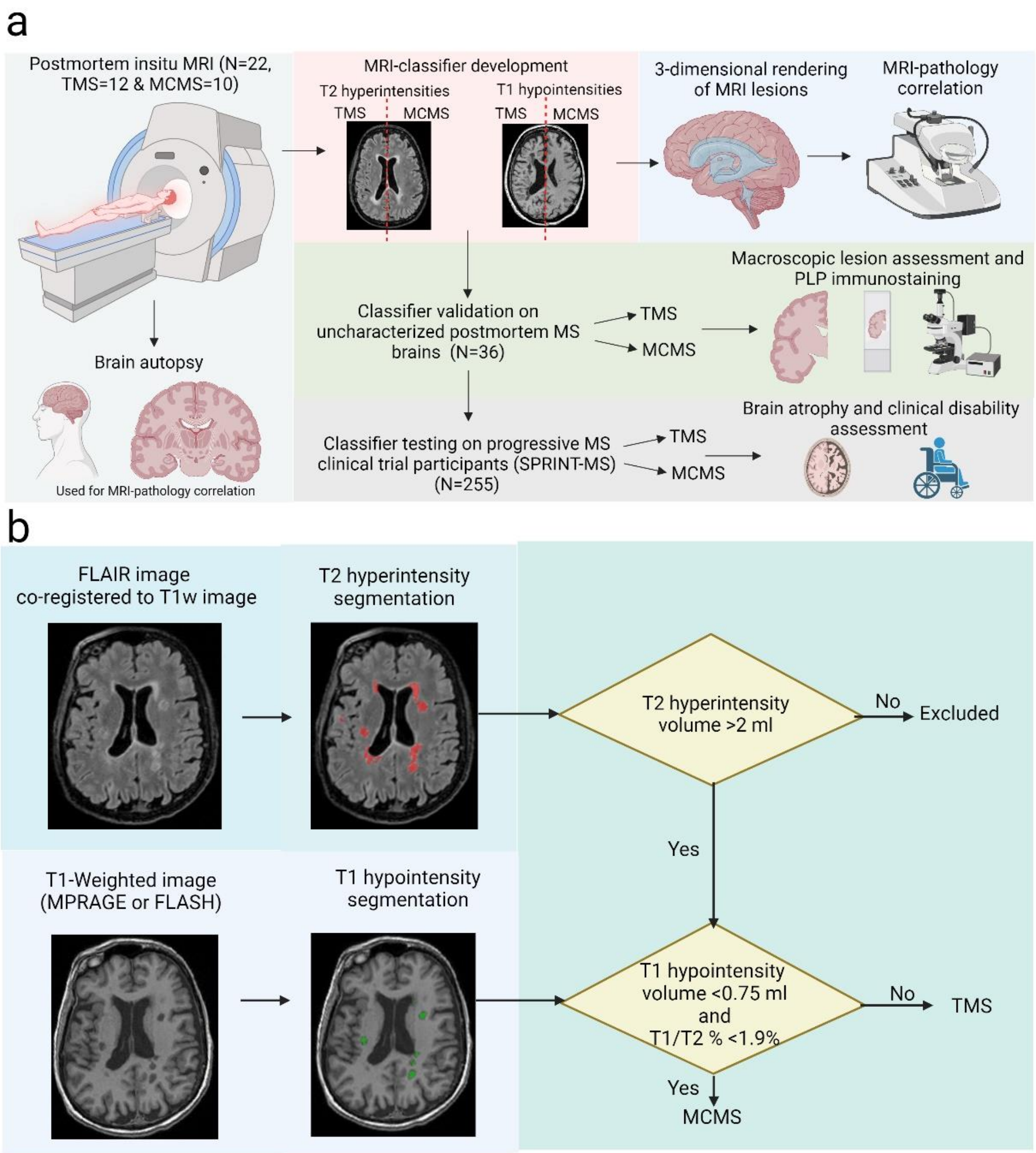

Figure S1 (**a**) Experimental workflow. (**b**) Flow chart of MRI classifier development.

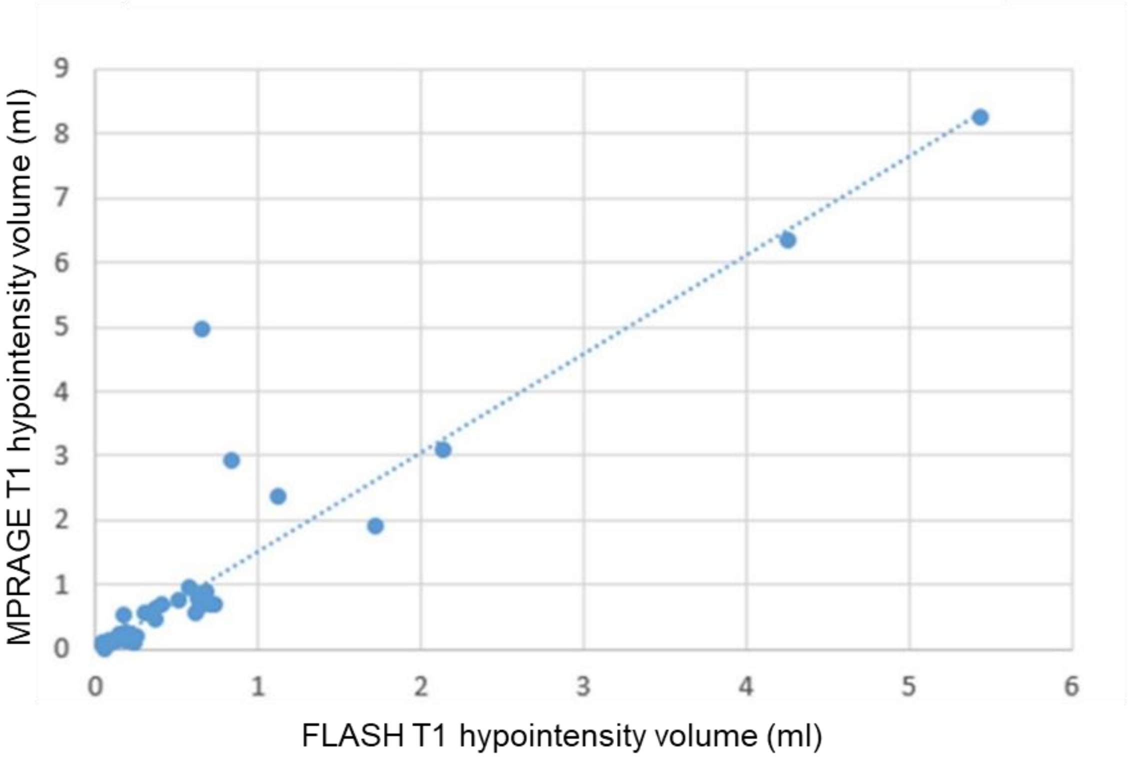

Figure S2 Calibration of T1 hypointensity volume across different MRI sequences.

a MCMS 7- Brain Slice 7
PLP immunostaining

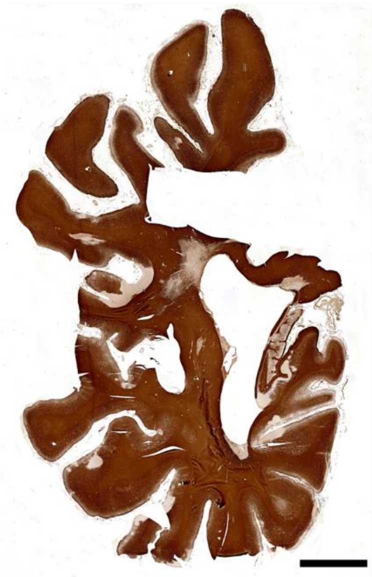

b WM macroscopic lesion area per hemisphere

MCMS 7		Area (cm^2)
Slice 5	Lesion 1	0.03
	Lesion 2	0.07
Slice 7	Lesion 1	0.07
	Lesion 2	0.37
	Lesion 3	0.08
	Lesion 4	0.13
	Lesion 5	0.03
Slice 9	Lesion 1	0.09
	Lesion 2	0.06
	Lesion 3	0.05
Slice 11	Lesion 1	0.15
	Lesion 2	0.09
Total WM hyperpigmented area		1.22

Figure S3 False positive MCMS postmortem brain.
a PLP immunostaining of hemispheric section confirms demyelinated WM lesions in false positive MCMS brain (*scale bar = 10 mm).* **b** Quantification of cerebral WM hyperpigmentation area in false positive MCMS brain slices.

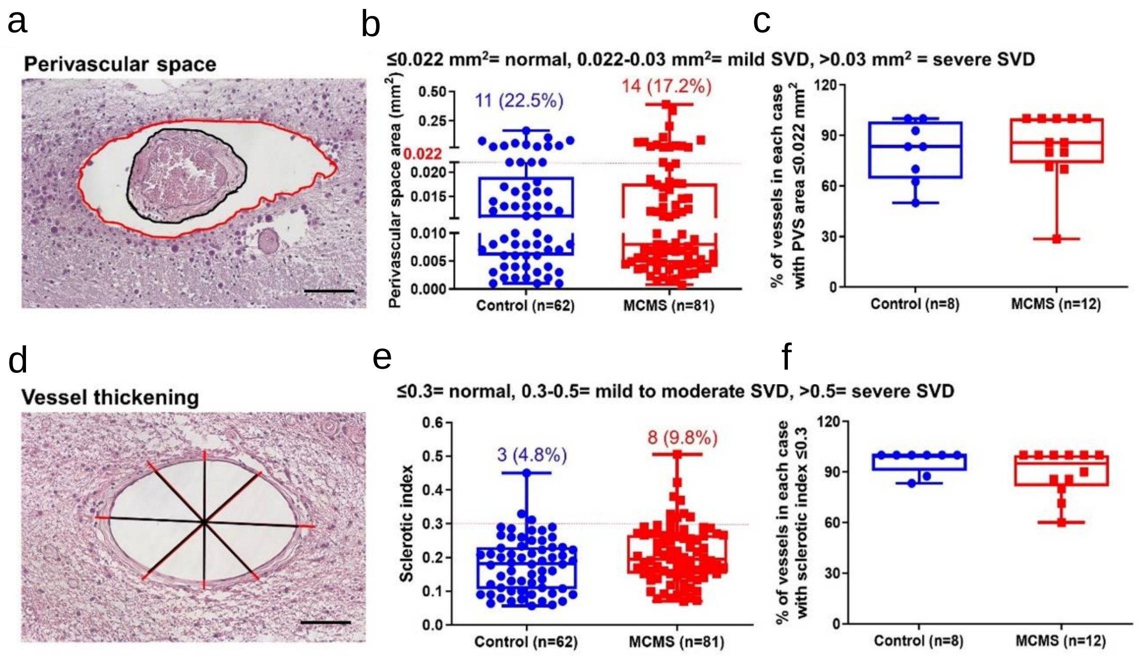

Figure S4 Small vessel disease assessment in MCMS periventricular WM.
a-f Comparison of perivascular space (**a, b and c**) and sclerotic index (**d, e and f**) of small vessels in control and MCMS periventricular WM sections stained with hematoxylin and eosin. Perivascular space area (**b and c**) and sclerotic index (**e and f**) of small vessels was similar in control and MCMS cases and neither measurement met the criteria (perivascular space area ≤0.022 mm^2; sclerotic index ≤0.3) for small vessel disease (*scale bar = 50 µm*).

Supplemental Table

Disability independent of cerebral white matter demyelination in progressive multiple sclerosis

Vikas Singh[1], Yufan Zheng[2], Daniel Ontaneda[3], Kedar R Mahajan[1,3], Jameson Holloman[1,3], Robert J Fox[3], Kunio Nakamura[2], and Bruce D Trapp[1]

Affiliations

[1]Department of Neurosciences, Lerner Research Institute, Cleveland Clinic, Cleveland OH

[2]Department of Biomedical Engineering, Lerner Research Institute, Cleveland Clinic, Cleveland OH

[3]Mellen Center for Treatment and Research in MS, Cleveland Clinic, Cleveland, OH

Correspondence to: Dr. Bruce D Trapp, PhD, NC30, Department of Neurosciences, Lerner Research Institute, Cleveland Clinic, 9500 Euclid Avenue, Cleveland, OH, 44195; trappb@ccf.org

Group	Case no.	MS Course	Sex	Ethnic group	Age at death (years)	Disease Onset	Disease Duration (years)	Initial Symptoms	Other clinical Symptoms	CSF results	Treatment	EDSS (Prior to Death)	PMI (hrs)	Cause of Death
MCMS	1	SP	F	- Black	60	7/01/1972	39.4	NA	Progressive paraparesis	NA	Pulse dose steroids	7	12	Choking (upper airway obstruction)
	2	SP	F	White	61	7/1/1996	18.5	Balance problems, diplopia	Leg weakness, worsening balance, fatigue	NA	Pulse dose steroids	8	11.5	End-Stage Renal Disease
	3	SP	F	White	54	7/1/1980	38.7	vision loss	Weakness, neurogenic bladder, cognitive loss, trigeminal neuralgia	NA	Avonex, Copaxone, IVSM	8	7.75	Respiratory arrest
	4	SP	F	White	61	7/1/1992	26.1	Left lower extremity numbness	Paraparesis, neurogenic bladder	OCB +	Avonex, Copaxone, Glatopa, Arava (RA), AZA (RA), MTX (RA)	8	8.75	Sepsis
	5	SP	F	Black	56	7/1/1980	35.5	NA	Paraplegia, neurogenic bladder, fatigue, vision loss	NA	Pulse dose steroids and oral steroids	8	13.5	Septic shock

	6	SP	F	White	52	7/1/1996	20.3	NA	Quadraparesis, neurogenic bladder, dysphagia, cognitive loss	OCB +	Copaxone, IV steroids, PO steroids	8	13.5	Respiratory failure
Abbreviations: CSF- cerebrospinal fluid; EDSS- Expanded Disability Status Scale; F- Female; IV- intravenous; M- Male; MCMS- Myelocortical Multiple Sclerosis; NA- Not available; OCB- Oligoclonal bands; PMI- Post-mortem Interval; SP- Secondary progressive.														

Table S1 Clinical details from myelocortical multiple sclerosis patients identified in postmortem validation cohort.
